# Lesson from a Single Pediatric Emergency Department: Potentially Applicable Radiation-Minimizing Practices for Non-Traumatic Abdominal Pain in Adolescents

**DOI:** 10.3390/children12101306

**Published:** 2025-09-28

**Authors:** Min Kyo Chun, Reenar Yoo, Soo-young Lim, Dahyun Kim, Jeeho Han, Seung Jun Choi, Jeong-Yong Lee, Jong Seung Lee, Jun Sung Park

**Affiliations:** 1 Department of Pediatrics, Asan Medical Center, University of Ulsan College of Medicine, Seoul 05055, Republic of Korea; 2 Department of Convergence Medicine, Asan Medical Center, Asan Institutes for Life Sciences, University of Ulsan College of Medicine, Seoul 05055, Republic of Korea; 3 Department of Emergency Medicine, Asan Medical Center, University of Ulsan College of Medicine, Seoul 05055, Republic of Korea

**Keywords:** adolescent, abdominal pain, point-of-care systems, emergency service, hospital

## Abstract

**Highlights:**

**What are the main findings?**

- Pediatric emergency department (PED) physicians used significantly fewer CT scans (28.5% vs. 37.9%) and fewer CT phases per patient (0.49 vs. 0.76 per patient) compared to adult ED physicians, while utilizing point-of-care ultrasound (POCUS) significantly more frequently (28.0% vs. 0.1%).
- Despite reduced radiation exposure and simplified imaging strategies (fewer CT phases and single-view X-rays), safety outcomes remained comparable between PED and AED.

**What is the implication of the main finding?**

- These findings support radiation-minimizing imaging practices in adolescents by highlighting the feasibility of POCUS as a frontline tool in the pediatric ED.
- Adoption of similar PED-style imaging strategies could reduce unnecessary radiation exposure in adolescents without compromising clinical outcomes.

**Abstract:**

Background/Objectives: In emergency departments (EDs), choosing imaging modalities for adolescents with abdominal pain requires balancing diagnostic accuracy and minimizing radiation exposure. This retrospective study compared imaging modalities in adolescents (16–18 years) presenting with non-traumatic acute abdominal pain between the pediatric ED (PED) and adult ED (AED) in the same institution. Methods: We conducted a retrospective study in patients aged 16–18 years who presented to AED or PED in the same tertiary university-affiliated hospital due to non-traumatic acute abdominal pain between January 2019 and July 2023 (study period = 55 months). The patient freely decided on the emergency department (ED) to be admitted. Results: This study analyzed 950 patients (683 in AED and 267 in PED). Actionable and surgical emergencies were comparable between both EDs (*p* = 0.617 and 0.245, respectively). PED physicians used fewer CT scans (28.5% vs. 37.9%, *p* = 0.006) and fewer CT phases (mean, 0.49 vs. 0.76, *p* < 0.001). Despite more patients undergoing X-rays in PED (77.9% vs. 61.6%, *p* < 0.001), the number of X-ray images was lower than in AED (mean, 0.9 vs. 1.1, *p* < 0.001). PED performed more point-of-care US (POCUS) than AED (28.0% vs. 0.1%, *p* < 0.001). Both EDs had comparable safety outcomes (revisits and missed surgical emergencies). Conclusions: PED physicians utilize POCUS more frequently and employ fewer CT scans, X-ray images, and CT phases than AED physicians in adolescents presenting with non-traumatic acute abdominal pain. Despite lower radiation exposure, the PED achieved safety outcomes comparable to the AED’s, indicating that a PED-style imaging strategy may be safely applied to adolescent abdominal pain evaluation.

## 1. Introduction

Ultrasound (US) is a more frequently used diagnostic modality than computed tomography (CT) in pediatric patients presenting to the emergency department (ED) with non-traumatic abdominal pain in the USA. When performed by skilled ED physicians or radiologists, Ultrasound (US) can achieve a sufficient diagnostic yield while minimizing radiation exposure [1,2]. Point-of-care US (POCUS) performed by ED physicians has been widely reported for its diagnostic value in pediatric surgical abdominal conditions, such as intussusception, appendicitis, and hypertrophic pyloric stenosis [3,4,5]. The increasing trend in the use of the US in pediatric patients aims to reduce radiation exposure, which has led to a gradual decrease in CT use [6,7,8,9].

However, the utilization of US in adolescents is not increasing as it is in younger pediatric age groups, and the rate of CT scans is not decreasing [10,11,12]. Hesitation in utilizing abdominal US in adolescents may be due to their relatively thicker abdominal wall resulting in suboptimal sonographic window, different disease prevalences that cannot be differentiated with US alone, and a lower reluctance to radiation exposure compared to younger children [13,14,15]. However, like in younger children, radiation exposure should also be considered critically in adolescents due to their higher sensitivity to radiation exposure and their longer life expectancy compared to adults as the dose in young children [11,12]. However, the utilization of US in adolescents is not increasing like in younger pediatric age groups, and the rate of CT scans is not decreasing [13,14,15]. Conversely, several studies have reported that CT utilization in adolescents has increased more rapidly than in other pediatric age groups [16,17]. Hesitation in utilizing abdominal US in adolescents may be due to their relatively thicker abdominal wall resulting in a suboptimal sonographic window, different disease prevalences that cannot be differentiated with US alone, and less reluctance to radiation exposure compared to younger children [18,19,20]. While the feasibility of POCUS in adolescents has been reported for select disease categories, studies focusing on abdominal POCUS utilization and associated radiation exposure in adolescents are scarce [21,22,23].

Kim et al. reported that adolescents aged 15–18 years with abdominal pain presenting to different EDs within the same institution, specifically, pediatric ED (PED) versus adult ED (AED), underwent fewer CT scans in PED [16]. This suggests a more conservative approach to CT utilization by PED physicians and simultaneously suggests that sufficient diagnostic discrimination can be achieved even with less radiation exposure in AED. However, their analysis lacked a comprehensive investigation of the frequency of POCUS and the detailed number of images or phases of X-rays and CT by ED physicians. This study aimed to fill this gap by analyzing the frequency and type of imaging modalities used in different EDs (AED vs. PED) within the same institution for adolescent patients with non-traumatic acute abdominal pain. In epidemiologically comparable cohorts, we compared the efficacy and risks of distinct radiologic testing strategies between two EDs and explored radiation-minimizing strategies that can be applicable to adolescents.

## 2. Materials and Methods

### 2.1. Population and Study Design

**Figure 1:**
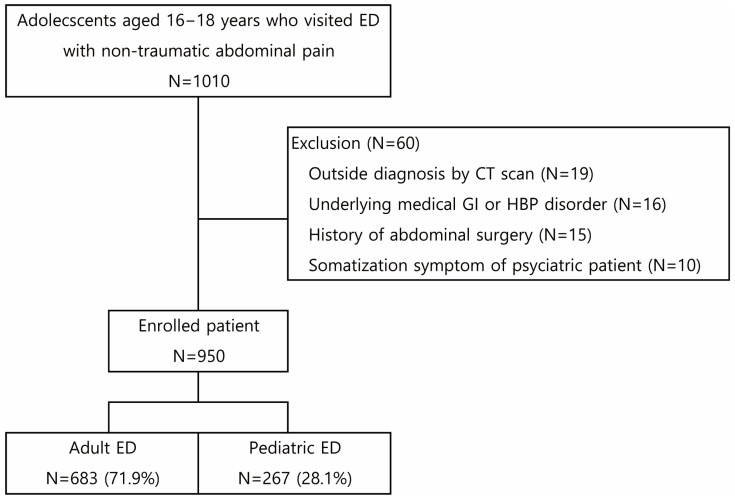
Flowchart of the study patient. Abbreviations: ED, emergency department; CT, computed tomography; GI, gastrointestinal; HBP, hepato-biliary-pancreatic.

We retrospectively enrolled patients aged 16–18 years who presented to a tertiary university-affiliated hospital ED due to acute abdominal pain between January 2019 and July 2023 (study period = 55 months). Because the institution’s medical records mandate the documentation of pain presence, location, and intensity, we included all patients with abdominal pain of ≤48 h’ onset, even when it was not the chief complaint. Patients with an injury event within 48 h, as defined by the Korean Triage and Acuity Scale (KTAS) [17], were classified as having traumatic abdominal pain and were excluded. Patients with underlying gastrointestinal or hepatobiliary conditions, abdominal surgery, suspected psychologic symptoms with pre-existing psychiatric diagnosis (e.g., somatization disorder, malingering, and Munchausen syndrome), and those being transferred after diagnosis with CT were also excluded from the study (Figure 1).

Because this was a retrospective study, patient allocation, imaging strategies, and standardized POCUS training could not be prespecified or assigned. Instead, we analyzed patients managed under existing routine practices. Details on patient recruitment and allocation, imaging selection, and the level of POCUS training are provided in the subsequent paragraphs.

The study institution has two EDs, AED and PED, located in separate building. The study affiliate institution evaluates approximately 120,000 and 35,000 patients annually in the AED and PED, respectively. The ED admission policy of the study affiliate is as follows: Patients aged ≤ 15 years should be admitted to the PED; patients aged between 16 and 18 years are free to choose among the two EDs, and patients aged ≥ 19 years should be admitted to the AED. In both EDs, the same triage system is applied according to the KTAS [17], which has the same standards for all patients over 15 years regardless of whether adult or adolescent. In both EDs, a fellow or higher-degree specialist performed the initial investigation, followed by a post-initial investigation by a resident. Final disposition and decisions are made by fellows, higher-degree specialists, or senior residents with at least three years of residency training. A dedicated attending physician is assigned to each ED, ensuring 24 h coverage and providing consultations to support clinical decision-making.

The choice of diagnostic modality is entirely at the physician’s discretion, as there is no established protocol for evaluating patients with abdominal pain. However, if a surgical emergency, such as ovarian torsion or acute appendicitis, is diagnosed on US or X-ray, a mandatory CT scan is required to confirm the diagnosis, assess accompanying complications, and evaluate anatomical variations—unless the patient is in shock and requires immediate surgical intervention. Both ED physicians have a similar level of POCUS training, including an annual half-day hands-on course. In both EDs, POCUS was readily available and usually performed for localized tenderness, peritoneal irritation, malignancy screening, severe pain (NRS ≥ 8), or suspected surgical abdomen, at the clinician’s discretion. All the discharged patients were counseled to revisit the ED if persistent or worsening abdominal pain occurs. The Institutional Review Board of the Asan Medical Center approved this study (IRB no. 2023-2757) and waived the requirement for informed consent due to the study’s retrospective nature.

### 2.2. Data Collection

Collected data included the clinical features (age, body weight, clinical severity according to KTAS, clinical presentations, pain scale, and physical examinations) and clinical course (diagnostic testing, length of stay, and disposition). Height was not a mandatory measurement. The numeric rating scale was used to assess the pain scale [18]. For X-rays and CTs, we investigated whether the test was performed, as well as the total number of images taken for X-ray and the number of phases taken for CT. If supine and erect X-ray images were taken together, it was counted as two, and if both pre- and post-contrast enhancement CT (CECT) phases were taken, it was counted as two. The final diagnosis was collected according to medical records from the discharge summary and outpatient follow-up. Among the study cohort, 813 (85.6%) patients were followed up and the final diagnosis was validated. A missed diagnosis of the other patients with follow-up loss was investigated through history taking in remote revisits. Every visit, including subsequent revisits, was enrolled as separate event. The actionable abdomen was defined as category one or two, requiring a clinical decision within minutes (category 1) or hours (category 2), as defined by the Actionable Reporting Work Group of the American College of Radiology [19].

### 2.3. Statistical Analysis

Ordered/count outcomes were summarized descriptively and compared nonparametrically where applicable. For between-group comparisons, continuous variables were summarized as mean ± SD or median (IQR) and analyzed using the Student’s *t*-test or Mann–Whitney U test, as appropriate following distributional assessment. Categorical variables were compared using the χ^2^ test when all expected cell counts were ≥5 (and <20% of cells had expected counts <5); otherwise, Fisher’s exact test was used. To evaluate the contribution of clinical factors to imaging modality selection, we performed multivariable logistic regression. These analyses were performed using SPSS Statistics for Windows, version 21.0. (IBM Corp., New York, NY, USA), where *p* < 0.05 was considered statistically significant.

## 3. Results

During the study period, 1010 patients aged 16–18 years visited the ED with non-traumatic acute abdominal pain. After excluding 60 patients, 950 were enrolled (Figure 1), including 683 (71.9%) and 267 (28.1%) patients who visited the AED and PED, respectively.

Table 1 summarizes patient demographic and clinical characteristics based on the visited ED.

Patients visiting PED had a lower proportion of males than those visiting AED (32.6% vs. 41.6%, *p* = 0.011) and had a mean younger age (16.6 years vs. 17.2 years, *p* < 0.001). Triage severity was higher in patients visiting PED than in those visiting AED (mean, 2.86 vs. 3.25, *p* < 0.001). Patients in PED reported higher pain scale scores (5 vs. 4, *p* < 0.001) and exhibited more localized tenderness (56.9% vs. 44.9%, *p* < 0.001) than those in AED. Similarly, patients visiting PED were more likely to require hospitalization than those visiting AED (24% vs. 13.5%, *p* < 0.001); however, there was no critical case such as intensive care unit admission or mortality in either group. The two groups had no significant difference regarding revisits or missed surgical emergency cases.

Both EDs had comparable proportions of actionable and surgical emergencies (*p* = 0.617 and 0.245, respectively, Table 2).

**Figure 2:**
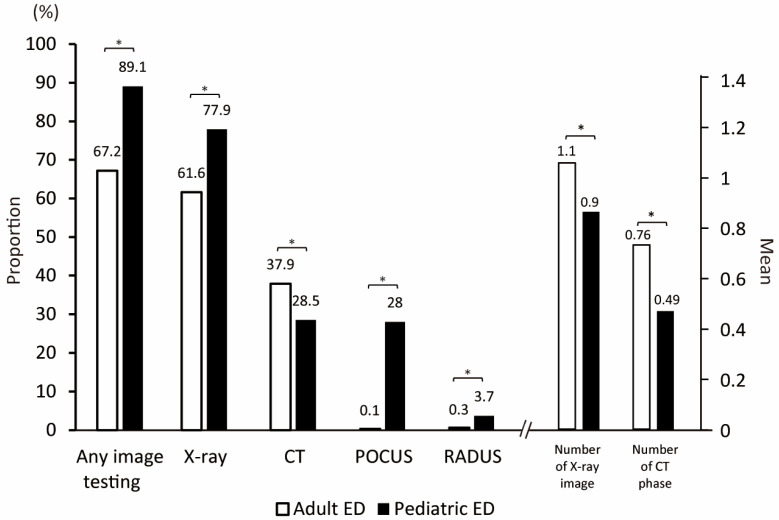
Diagnostic work-up during emergency department stay. * *p* < 0.001. Abbreviations: ED, emergency department; CT, computed tomography; POCUS, point-of-care ultrasound; RADUS, radiologist-performed ultrasound.

Except for acute pyelonephritis (3% vs. 1%, *p* = 0.024), the two EDs did not differ in the prevalence of individual diseases. Table 3 and Figure 2 summarize the diagnostic tests conducted in both EDs.

Compared to AED, PED more frequently utilized any imaging modality (89.1% vs. 66.8%, *p* < 0.001). The rate of X-ray imaging was higher in PED (77.9% vs. 61.6%, *p* < 0.001), although the total number of images was lower (mean, 0.9 vs. 1.1, *p* < 0.001) compared to those in AED. Among the patients who underwent X-rays, PED physicians tended to take a single image (58.7% with supine and 29.8% with erect), while AED physicians took both supine and erect X-rays (86.9%). PED had a lower rate of CT scans (28.5% vs. 37.9%, *p* = 0.006) and fewer total number of phases (mean, 0.49 vs. 0.76, *p* < 0.001) compared to AED. Among the patients who underwent CT, a single phase of CECT was mainly performed in PED (89.5%), while both non-contrast-enhanced CT (NECT) and CECT were performed together in AED (96.1%). The utilization rates of US (both POCUS and radiologist-performed US) were higher in PED (28.0% vs. 0.1%, *p* < 0.001 and 3.7% vs. 0.3%, *p* < 0.001, respectively). In the multivariate analysis conducted to adjust for differences in patient characteristics between AED and PED (Table 4), the ED site was found to influence POCUS use (odds ratio = 25.8, and *p* = 0.003, respectively). Other imaging tests and patient disposition were not influenced by the ED site.

**Figure 3:**
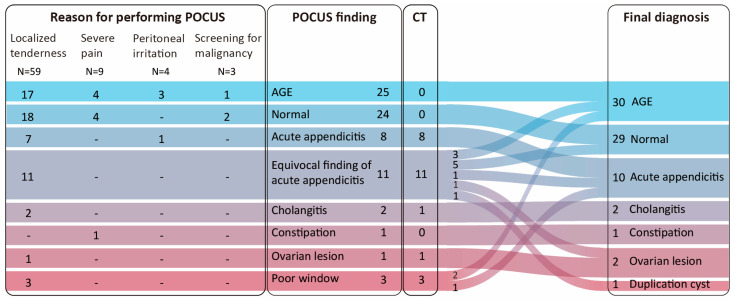
Performance of point-of-care ultrasound in pediatric emergency department. Abbreviations: POCUS, point-of-care ultrasound; CT, computed tomography; AGE, acute gastroenteritis.

Localized tenderness was the most common reason for performing POCUS (N = 59, 78.7%, Figure 3).

POCUS findings identified acute gastroenteritis (AGE) in 25 (33.3%) cases, normal findings in 24 (32%) cases, and acute appendicitis in eight (10.7%) cases. Among the 24 patients (32%) who underwent subsequent CT scans, 9were for mandatory confirmation of a suspected surgical emergency identified by POCUS (8 with appendicitis, 1 with ovarian cyst rupture) Another 14 scans were performed for incomplete POCUS studies (11 with equivocal finding of appendicitis, three with poor window), and 1 scan was to evaluate for obstructive lesion in a case of acute cholangitis. Among 11 patients with equivocal findings of acute appendicitis (e.g., normal appendix diameter with sonographic tenderness or the opposite), 3 were diagnosed with AGE, 5 with normal findings, 1 with acute appendicitis, 1 with ovarian cyst rupture, and 1 with a duplication cyst. Of the three patients with poor window were diagnosed AGE in two patients and appendicitis in one patient upon CT.

Overall screening performance for actionable and surgical abdomen is presented in Table 5. Sensitivities were 93.3% and 91.7% for actionable and surgical abdomen, respectively; negative predictive values were 98.1% and 98.2%; and accuracies were 83.3% and 85.9%, respectively.

## 4. Discussion

Our research found that among adolescents aged 16–18 years with non-traumatic acute abdominal pain, the PED conducted fewer CT scans and more POCUS compared to the AED, despite a comparable prevalence of actionable and surgical emergencies. Although CT scans were reduced in the PED, the safety outcomes (revisit rate and missed surgical abdomen) were comparable to those in the AED. In the PED, a single supine image was predominantly used for X-ray imaging, and a single-phase CECT was mainly used for CT imaging. Conversely, the AED predominantly used two X-ray images and two phases of CT.

Continuous concern regarding radiation and efforts to minimize it have led to a trend in the ED to reduce CT scans and increase the use of US (e.g., Image Gently campaign) [12,20,21,22]. Especially among PED physicians who primarily treat pediatric patients who are more sensitive to radiation exposure and have longer life expectancy, efforts have been made to substitute some CT with POCUS [7,10,11,12,20]. In this study, PED physicians utilized POCUS significantly more than AED physicians and employed fewer CT scans. Despite a higher prevalence of localized tenderness indicating the possibility of surgical emergencies in patients undergoing POCUS, the rate of CT scan was similar to that of patients who did not undergo POCUS, and the rate of X-ray imaging was even lower (Appendix A Table A1). This increased reliance on POCUS, likely driven by the PED physicians’ reluctance to use radiation, contributed to a reduction in radiation exposure.

Among the 14 PED cases of acute appendicitis, the most common surgical emergency in adolescents, eight (57.1%) cases were diagnosed using POCUS alone. Several studies have reported a decrease in CT usage with an increase in POCUS utilization in pediatric appendicitis [5,23,24]. Beyond appendicitis, evidence supporting POCUS is accumulating across a range of pediatric emergency conditions, and similar patterns may extend to additional diagnoses [10,25,26]. However, direct comparative data on the effect of ultrasound implementation on radiation reduction is largely disease-specific, and we identified few studies that span multiple diagnoses in adolescents [24,27]. By evaluating all adolescent abdominal pain presentations at the point of diagnostic uncertainty in the ED workflow, our study provides service-level support for a POCUS-friendly, radiation-minimizing approach with comparable safety outcomes.

In the PED, supine X-rays were primarily performed alone, while in CT, only one phase of CECT is commonly performed. In the AED, both additional erect image and NECT tended to be performed together. However, there was no significant difference in safety outcomes (missed cases or revisits) between the two EDs. Several studies have reported that concurrent erect abdominal X-ray provides few additional clinical benefits compared to supine X-ray alone in patients with acute abdomen [28,29,30,31]. Similarly, although CECT and NECT have unique advantages, performing CECT alone does not compromise clinical decisions compared to performing both in several conditions [32,33]. Furthermore, CECT is known to have 30% higher accuracy than NECT in acute abdomen patients [34]. Even in urinary stones, of which NECT is known to have a representative advantage, CECT is sufficient for detection when the size of the stone exceeds 3–6 mm, which is challenging to pass naturally [35,36]. Therefore, our results suggest that a radiation-minimizing strategy omitting unnecessary routine erect imaging or NECT phases may not be inferior for patients with acute abdominal pain.

There is still room for improvement in the PED’s radiation-minimizing effort. Although the number of X-ray images per patient was lower, the proportion of patients undergoing X-rays was higher than in the AED. Considering stochastic effects other than the deterministic effect of diagnostic radiation, even small amounts of X-ray radiation exposure may pose a potential cancer risk [37,38,39,40]. Therefore, efforts should be made to minimize not only the total number of images but also the number of patients undergoing even a single unnecessary X-ray. In addition, since POCUS in this study was used based on individual physicians’ clinical decisions rather than specific indications, it is unclear whether the same benefits would be observed in other patients. This is true even though both patient groups had similar demographic features. Among the 75 patients who underwent POCUS, 14 (18.7%) showed equivocal or incomplete findings, and 2 were diagnosed with acute appendicitis on subsequent CT scans. Therefore, despite its radiation-sparing capacity, abdominal POCUS should still be utilized only as a pre-imaging screening tool before CT, not as a replacement [5,41].

A key consideration in this study was the significant differences in patient characteristics, such as age, sex, and clinical manifestations, between the two EDs, despite the random selection of ED and similar prevalence of surgical emergencies. However, multivariate analysis controlling for these clinical factors found that POCUS utilization was independently associated with the ED section, while patient disposition was not. This finding suggests that differences in POCUS use were primarily due to variations in physician practice styles rather than patient characteristics. Since physicians in both EDs had similar training backgrounds, the increased reliance on POCUS in the PED likely reflects their greater familiarity with evaluating pediatric abdominal symptoms, which they encounter more frequently in daily practice.

This study has several limitations. First, as mentioned earlier, due to the study’s retrospective nature, there was no complete random assignment, making it impossible to correct this bias. Additionally, being a single-center study, it is challenging to generalize the findings to populations with different demographics or varying disease incidences. Moreover, without predetermined indications for performing POCUS or recorded reasons for proceeding directly to CT, the diagnostic yield of POCUS in acute abdominal pain could not be accurately evaluated Therefore, the comparison was limited to assessing the familiarity of two ED physicians with POCUS. Although data collection for missed surgical emergencies during outpatient follow-up or remote revisit was feasible for most patients, comprehensive follow-up was not achieved. Given these limitations, future multicenter, prospective investigations incorporating random allocation, predefined POCUS indications, standardized training requirements, and imaging protocols are warranted to confirm the clinical utility of POCUS in adolescents with acute abdominal pain and to substantiate the PED’s radiation-minimization efforts.

## 5. Conclusions

PED physicians utilize POCUS more frequently and employ fewer CT scans, X-ray images, and CT phases than AED physicians in adolescents presenting with non-traumatic acute abdominal pain. Despite lower radiation exposure, the PED achieved safety outcomes comparable to the AED’s, indicating that a PED-style imaging strategy may be safely applied to adolescent abdominal pain evaluation.

## Figures and Tables

**Table 1:** Demographic and clinical characteristics of the study patient.

		Adult ED	Pediatric ED	*p*-Value
		N = 683	N = 267	
Sex, male		284 (41.6)	87 (32.6)	0.011
Age, year				
	Mean (95% CI)	17.2 (17.1–17.2)	16.6 (16.5–16.7)	<0.001
	16	174 (25.5)	146 (54.7)	NA
	17	225 (32.9)	89 (33.3)	
	18	284 (41.6)	32 (12)	
Body weight, kg		58 (51.3–66.5)	56 (49–65.5)	0.124
KTAS				
	Mean (95% CI)	3.25 (3.22–3.29)	2.86 (2.79–2.93)	<0.001
	1	1 (0.1)	2 (0.7)	NA
	2	13 (1.9)	61 (22.8)	
	3	484 (70.9)	177 (66.3)	
	4	182 (26.6)	26 (9.7)	
	5	3 (0.4)	1 (0.4)	
Clinical manifestation				
	Pain scale	4 (3–4)	5 (3–7)	<0.001
	Vomiting	238 (34.8)	91 (34.1)	0.843
	Diarrhea	263 (38.5)	71 (26.6)	0.001
	Tenderness	307 (44.9)	171 (64.0)	<0.001
	Rebound tenderness	24 (3.5)	9 (3.4)	0.242
Disposition				
	Discharge	587 (85.9)	202 (75.7)	<0.001
	Need for admission	92 (13.5)	64 (24.0)	0.001
	Admission	27 (4)	56 (21)	<0.001
	ICU admission	0 (0)	0 (0)	NA
	Transfer	65 (9.5)	8 (3.0)	0.001
	Mortality	0 (0)	0 (0)	NA
ED LOS, hour		3.2 (1.9–5.2)	4 (2.5–7.5)	<0.001
Revisit within 2 days		19 (2.8)	14 (5.2)	0.061
Missed surgical emergency		5 (0.7)	1 (0.4)	NA
	Acute appendicitis	4 (0.6)	1 (0.4)	NA
	Ovarian torsion	1 (0.1)	0 (0)	NA

Abbreviations: ED, emergency department; CI, confidence interval; KTAS, Korean triage and acuity scale; ICU, intensive care unit; LOS, length of stay; NA, not applicable. Values are represented as number (%) or median (interquartile range).

**Table 2:** Diagnosis of the study patients.

			Adult ED	Pediatric ED	*p*-Value
			N = 683	N = 267	
Actionable emergency			116 (17)	49 (18.4)	0.617
	Surgical emergency		54 (7.9)	18 (6.7)	0.245
		Acute appendicitis	39 (5.7)	14 (5.2)	0.778
		Ovarian torsion	9 (1.3)	0 (0)	0.068
		Panperitonitis	2 (0.3)	0 (0)	NA
		Pancreatic tumor rupture	1 (0.1)	0 (0)	NA
		Sigmoid volvulus	1 (0.1)	0 (0)	NA
		Splenic infarction	1 (0.1)	0 (0)	NA
		Stomach perforation	1 (0.1)	0 (0)	NA
		Duplication cyst	0 (0)	1 (0.4)	NA
		Bowel obstruction due to lymphoma	0 (0)	1 (0.4)	NA
		Venous malformation rupture	0 (0)	1 (0.4)	NA
		Colonic perforation	0 (0)	1 (0.4)	NA
	Cancer		10 (1.5)	6 (2.2)	0.399
	Urinary stone		9 (1.3)	2 (0.7)	0.737
	Ovarian cyst rupture		7 (1)	0 (0)	0.200
	Biliary stone		1 (0.1)	5 (1.9)	NA
	Pancreatitis		2 (0.3)	2 (0.7)	NA
	Superior mesenteric artery syndrome		2 (0.3)	0 (0)	NA
	Unexpected pregnancy		1 (0.1)	0 (0)	NA
	Infection				
		Acute pyelonephritis	7 (1)	8 (3)	0.024
		Hepatitis	6 (0.9)	2 (0.7)	1.000
		Cholangitis	2 (0.3)	3 (1.1)	NA
		Diverticulitis	5 (0.7)	0 (0)	NA
		Cholecystitis	3 (0.4)	0 (0)	NA
		Intraabdominal abscess	2 (0.3)	1 (0.4)	NA
		Pelvic inflammatory disease	2 (0.3)	0 (0)	NA
		Epiploic appendicitis	1 (0.1)	0 (0)	NA
		Extra-abdominal infection ^†^	2 (0.3)	2 (0.7)	NA
Acute gastroenteritis			395 (57.8)	135 (50.6)	0.096
Non-specific abdominal pain			130 (19)	59 (22.1)	0.211
Others ^‡^			42 (6.1)	24 (9)	NA

^†^ Extra-abdominal infection includes myocarditis and pneumonia. ^‡^ Others include inflammatory bowel disease, anaphylaxis, vasovagal syncope, rhabdomyolysis, diabetic ketoacidosis, constipation, dysmenorrhea, simple ovarian cyst, and peptic ulcer disease. NA, not applicable. Values are represented as numbers (%).

**Table 3:** Diagnostic work-up during emergency department stay.

				Adult ED	Pediatric ED	*p*-Value
				N = 683	N = 267	
Any image modality				459 (67.2)	238 (89.1)	<0.001
	X-ray					
		Mean (95% CI) number of images		1.1 (1.08−1.22)	0.9 (0.83−0.98)	<0.001
		Any		421 (61.6)	208 (77.9)	<0.001
			Supine only *	10 (2.4)	122 (58.7)	<0.001
			Erect only *	46 (10.9)	62 (29.8)	<0.001
			Both *	366 (86.9)	28 (13.5)	<0.001
	CT					
		Mean (95% CI) number of phases		0.76 (0.69−0.83)	0.49 (0.24−0.36)	<0.001
		Any		259 (37.9)	76 (28.5)	0.006
			NECT only *	11 (4.2)	4 (5.3)	1.000
			CECT only *	0 (0)	68 (89.5)	<0.001
			Both *	249 (96.1)	4 (5.3)	<0.001
	POCUS			1 (0.1)	75 (28.0)	<0.001
	RADUS			2 (0.3)	10 (3.7)	<0.001

Values are presented as number (%). Abbreviations: ED, emergency department; CI, confidence interval; CT, computed tomography; POCUS, point-of-care ultrasound; RADUS, radiologist-performed ultrasound. * Percentages and *p* value were calculated based on the number of patients who underwent testing (X-ray or CT).

**Table 4:** Multivariate Analysis of Imaging Test Selection and disposition.

Imaging Tests	Variables	B	SE	Wals	*p* Value	OR
Any image testing	Intercept	1.13	0.23	23.0	<0.001	3.1
X-ray	Tenderness	1.55	0.50	9.6	0.002	4.7
	Intercept	0.32	0.33	0.9	0.332	1.4
POCUS	PED (vs. AED)	3.25	1.10	8.8	0.003	25.8
	Tenderness	1.98	0.89	5.0	0.025	7.3
	Intercept	−3.51	1.08	10.5	0.001	0.03
CT	Vomiting	−1.49	0.62	5.7	0.017	0.23
	Tenderness	1.81	0.56	10.4	0.001	6.1
	Intercept	−1.69	0.49	12.0	0.001	0.18
Admission required	Rebound tenderness	2.98	1.08	7.6	0.006	19.73
	Tenderness	4.34	0.89	23.8	0.000	76.4
	Intercept	−3.62	0.72	25.5	0.000	0.03

Abbreviations: SE, standard error; OR, odds ratio; PED, pediatric emergency department; AED, adult emergency department; POCUS, point-of-care ultrasound; CT, computed tomography.

**Table 5:** POCUS screening performance for actionable and surgical abdomen.

	Sensitivity	Specificity	PPV	NPV	Accuracy
Actionable abdomen	93.3	81.0	53.8	98.1	83.3
Surgical abdomen	91.7	84.8	52.4	98.2	85.9

Values are presented as percent (%).

## Data Availability

The datasets generated during and/or analyzed during the current study are not publicly available due to the institutional review board of the Asan Medical Center, Seoul, Korea (IRB no. 2023-2757) not allowing sharing with out-of-hospital facility because of ethical consideration but are available from the corresponding author on reasonable request.

## Abbreviations

The following abbreviations are used in this manuscript:

ED	Emergency Department
PED	Pediatric ED
AED	Adult ED
CT	Computed Tomography
US	Ultrasound
POCUS	Point-Of-Care Ultrasound
KTAS	Korean Triage And Acuity Scale
CECT	Contrast-Enhanced CT
NECT	Non-Contrast-Enhanced CT
AGE	Acute Gastroenteritis
AJR	American Journal Of Roentgenology
ACR	American College Of Radiology
GI	Gastrointestinal
HBP	Hepatobiliary-Pancreas
RADUS	Radiologist-Performed Ultrasound

## Appendix A

**Table A1:** Patient characteristics according to performing point-of-care ultrasound in the pediatric emergency department.

		POCUS Not Performed	POCUS Performed	*p*-Value
		N = 192	N = 75	
Sex, male		62 (32.3)	25 (33.3)	0.870
Age, year		16 (16–17)	16 (16–17)	0.723
	Mean (95% CI)	16.6 (16.5–16.7)	16.5 (16.4–16.7)	0.638
	16	105 (54.7)	41 (54.7)	NA
	17	61 (31.8)	28 (37.3)	
	18	26 (13.5)	6 (8)	
Body weight, kg		56 (49–66)	56 (50–65)	0.644
KTAS				
	Mean (95% CI)	2.84 (2.76–2.93)	2.91 (2.78–3.04)	0.079
	1	1 (0.5)	1 (1.3)	NA
	2	47 (24.5)	14 (18.7)	
	3	126 (65.6)	51 (68)	
	4	17 (8.9)	9 (12)	
	5	1 (0.5)	0 (0)	
Clinical manifestation				
	NRS	5 (3–7)	4 (3–6)	0.165
	Localized tenderness	104 (54.2)	67 (89.3)	<0.001
	Rebound tenderness	5 (2.6)	4 (5.3)	NA
Any radiation exposure		152 (79.2)	58 (77.3)	0.742
	X-ray	157 (82.6)	51 (68)	0.028
	CT	52 (27.1)	24 (32)	0.423
Disposition				
	Discharge	146 (76)	56 (74.7)	0.814
	Need for admission	45 (23.4)	19 (25.3)	0.744
	Admission	42 (21.9)	14 (18.7)	0.563
	ICU admission	0 (0)	0 (0)	NA
	Transferred	3 (1.6)	5 (6.7)	0.028
	Mortality	0 (0)	0 (0)	NA
ED LOS, min		239 (157–451)	223 (127–457)	0.546
Revisit within two days		13 (6.8)	1 (1.3)	0.071
Missed surgical abdomen		1	0 (0)	NA
	Acute appendicitis	1	0 (0)	NA

Values are presented as number (%) or median (interquartile range). Abbreviations: ED, emergency department; POCUS, point-of-care ultrasound; CI, confidence interval; KTAS, Korean triage and acuity scale; NRS, numerical rating scale; CT, computed tomography; ICU, intensive care unit; LOS, length of stay; NA, not applicable.
